# Inflammatory Bowel Disease and Sarcopenia: Its Mechanism and Clinical Importance

**DOI:** 10.3390/jcm10184214

**Published:** 2021-09-17

**Authors:** Hiroki Nishikawa, Shiro Nakamura, Takako Miyazaki, Kazuki Kakimoto, Shinya Fukunishi, Akira Asai, Shuhei Nishiguchi, Kazuhide Higuchi

**Affiliations:** 1 The Second Department of Internal Medicine, Osaka Medical and Pharmaceutical University, Takatsuki 569-8686, Japan; saab460@gmail.com (S.N.); takako.miyazaki@ompu.ac.jp (T.M.); kazuki.kakimoto@ompu.ac.jp (K.K.); shinya.fukunishi@ompu.ac.jp (S.F.); in2108@osaka-med.ac.jp (A.A.); kazuhide.higuchi@ompu.ac.jp (K.H.); 2 The Premier Departmental Research of Medicine, Osaka Medical and Pharmaceutical University, Takatsuki 569-8686, Japan; 3 The Department of Internal Medicine, Kano General Hospital, Osaka 531-0041, Japan; nishiguchi@heartfull.or.jp

**Keywords:** inflammatory bowel disease, sarcopenia, mechanism, outcome, intervention

## Abstract

Malnutrition is a major contributor to muscle loss and muscle dysfunction, known as sarcopenia. Malnutrition is common in patients with inflammatory bowel disease (IBD). IBD includes ulcerative colitis (UC) and Crohn’s disease (CD). The number of patients with IBD has recently been increasing. More severe malnutrition is often seen in CD compared to UC, probably due to CD affecting the main site of nutrient absorption, extensive mucosal lesions, fistulas, short bowel syndrome after resection, or obstruction of the gastrointestinal tract. A recent meta-analysis showed the high prevalence of sarcopenia in patients with IBD, and thus sarcopenia is a very important problem for IBD. Although IBD is more common in younger patients, sarcopenia can develop through a variety of mechanisms, including malnutrition, chronic inflammation, increased inflammatory status in adipose tissue, vitamin deficiency, and imbalance of the muscle–gut axis. In addition, sarcopenia has a negative impact on postoperative complications and hospital stay in patients with IBD. Appropriate intervention for sarcopenia may be important, in addition to clinical remission and endoscopic mucosal healing in patients with IBD. Much more attention will thus be paid to sarcopenia in patients with IBD. In this review, we outline IBD and sarcopenia, based on the current evidence.

## 1. Introduction

Malnutrition is a major contributor to muscle loss and muscle dysfunction, known as sarcopenia, and it is defined as “a condition with altered body composition, impaired physical performance, and poor clinical outcome, due to inadequate nutrient intake or uptake” [1]. Malnutrition is common in patients with inflammatory bowel disease (IBD) [1]. Sarcopenia was first reported by Rosenburg in 1989 and defined as “aging-related loss of muscle mass” [2]. However, it has been shown that sarcopenia can also develop as a result of malignancies, malnutrition, inactivity, infection, and chronic inflammation (i.e., secondary sarcopenia) [3]. In the recent Asian Working Group for Sarcopenia (AWGS) and European Working Group of Sarcopenia in Older People (EWGSOP) definitions, loss of muscle mass accompanied by loss of muscle strength and physical performance is judged to be severe sarcopenia [4,5].

IBD includes ulcerative colitis (UC) and Crohn’s disease (CD). UC is an idiopathic, chronic inflammatory disease that affects the colonic mucosa continuously, from the rectum to the colon [6,7]. CD, on the other hand, is a progressive inflammatory disease that involves all layers of the gastrointestinal (GI) tract and can occur in any part of the GI tract, with complications such as stenosis, fistulae, and abscess occurring at a higher rate than UC [8,9]. IBD is an acute and chronic disease with exacerbations and remissions, and there is still no curative therapy for CD. It is widely known that CD shows a progressive tendency for decline in GI function through the formation of GI complications, such as severe stenosis, fistulae, and abscesses, during its clinical course, with repeated relapses and remissions, resulting in a high rate of indication for intestinal resection [8,9].

It is not uncommon for activity of daily life (ADL) to decline due to resistance to treatment in patients with CD [8,9]. Due to these characteristics of the clinical course of CD, control of the activity of CD alone is not sufficient as a therapeutic goal. The importance of achieving mucosal healing earlier in the course of the disease has been shown to correlate with an improved long-term prognosis [8,9]. UC, on the other hand, can be cured by total colonic resection, but it is important to maintain the remission stage with appropriate pharmacological intervention [6,7]. It is also important to avoid systemic administration of corticosteroids, as much as possible with adequate 5-aminosalicylic acid (5-ASA) preparations. For refractory patients with UC, early intervention with appropriate alternative therapies is required to wean off corticosteroids, rather than continuing steroid therapy indiscriminately as a basic treatment strategy [6,7]. Corticosteroids promote protein catabolism in skeletal muscle by the crosstalk between the glucocorticoid receptor and mechanistic target of the rapamycin complex 1 (mTORC1) [10]. mTORC1 acts not only to promote anabolism, but also to inhibit catabolism [10].

The number of patients with IBD has been increasing in recent years [11]. In 2014, the annual prevalence rates of UC and CD per 100,000 persons were 172.9 (192.3 in males and 154.5 in females) and 55.6 (79.5 in males and 33.1 in females), respectively [11]. As mentioned earlier, the basic treatment of UC is pharmacotherapy, and nutritional therapy itself does not lead to the remission stage of UC. The treatment of CD, on the other hand, involves a two-pronged approach: pharmacotherapy and nutritional therapy [6,7,8,9]. Intravenous nutrition in CD is expected to have an anti-inflammatory effect, by not exposing the GI tract to dietary antigens and fats [8]. If the Crohn’s disease is highly active and the nutritional status is markedly deteriorated, fasting is required for its management, and total parenteral nutrition is usually indicated [8,9].

More severe malnutrition is often seen in CD compared to UC [12]. This may be due to CD affecting the main site of nutrient absorption, extensive mucosal lesions, fistulas, short bowel syndrome after resection, or obstruction of the GI tract [12]. In CD, cytokines, such as interleukin-6 (IL-6), which are directly involved in the pathogenesis of the disease, inhibit the albumin promoter and induce hypoalbuminemia or act on the iron metabolism, resulting in anemia [8]. On the other hand, recent remarkable advances in therapeutic modalities for patients with IBD, such as antibody therapy and molecular targeted therapies, which focuses on inhibiting the specific molecular targets associated with inflammation of the intestinal mucosa, have been made, while the prevalence and prognostic impact of sarcopenia in IBD has not received much attention. A recent meta-analysis showed that 52% of patients with CD and 37% of patients with UC have sarcopenia [13], while sarcopenia seems to be a very important problem for patients with IBD. In patients with ankylosing spondylitis, which is an occasional complication of IBD, 34.3% were reported to have sarcopenia [14]. In addition, sarcopenia has a negative impact on postoperative complications and hospital stay in patients with IBD [15]. At the end of 2020, Selecting Therapeutic Targets in Inflammatory Bowel Disease (STRIDE)-II was published, which advocates not only clinical remission and mucosal healing as treatment goals for patients with IBD, but also the avoidance of structural destruction and dysfunction of the intestinal tract, as well as improvement of physical function, as long-term treatment goals [16]. These statements suggest that sarcopenia is a very important condition in patients with IBD. In this review, we will outline IBD and sarcopenia based on the current evidence.

## 2. Methods and Frequency of Sarcopenia Assessment in Patients with IBD

Table 1 shows the evaluation methods and frequency of sarcopenia reported in recent years in patients with IBD [17,18,19,20,21,22,23,24,25,26,27,28,29,30,31,32]. In more than half of the reports, computed tomography (CT, L3 level) is used to assess sarcopenia, but many reports only use muscle mass assessment to determine sarcopenia, while a few include grip strength and walking speed in the assessment of sarcopenia [17]. Furthermore, unlike primary sarcopenia [4,5], no reference values have been established for muscle mass, grip strength, or walking speed in patients with IBD. Moreover, screening tools for sarcopenia such as SARC-F (a questionnaire consisting of five questions) as recommended by AWGS and EWGSOP, are not available in patients with IBD [4,5,33]. On the other hand, the usefulness of skeletal muscle signal intensity in magnetic resonance imaging (MRI) T1-weighted images for the clinical outcomes in patients with CD has also been reported [34]. It has been reported that muscle mass in MRI correlates well with lean body mass using a bioelectrical impedance analysis (BIA) method in pediatric patients with UC [35]. The frequency of sarcopenia in patients with IBD varies from report to report, ranging from about 20–70%, as shown in Table 1. This may be due to differences in the definition of sarcopenia and differences in body size between Western and Asian patients with IBD. In acute severe UC, the frequency of sarcopenia was reported to be 70% [23]. In 29 pediatric patients with UC, 62% were reported to have sarcopenia [26].

## 3. Mechanisms of Sarcopenia in Patients with IBD

### 3.1. Malabsorption

Malabsorption syndrome is a condition caused when nutrients in the food are not properly absorbed in the small intestine for various reasons and is directly related to the development of sarcopenia [36]. IBD is one of the most common diseases that cause malabsorption [37]. Increased inflammation in the gut releases inflammatory mediators such as TNFα and IL-6, which increase intestinal permeability and cause local and systemic inflammatory effects [36,38]. Intestinal inflammation shortens the contact time between nutrients and the intestinal mucosal surface, exacerbating malabsorption and reducing the absorption of amino acids [36]. Since amino acids are a major anabolic signal in muscle, decreased amino acid absorption will induce sarcopenia [38]. In particular, leucine deficiency causes a significant reduction in muscle protein synthesis [39]. In a recent randomized controlled trial (RCT), the benefit of leucine supplementation on sarcopenia in the elderly was reported [39]. On the other hand, resection of the intestine reduces the surface area of the mucosa that can absorb nutrients, resulting in malnutrition [37]. Vomiting, diarrhea, anorexia, appetite loss, and side effects of medications (e.g., pancreatitis) can also cause malabsorption [37]. Medications for IBD can cause pancreatitis, especially thiopurines and mesalazine [40]. Trace element deficiencies due to malabsorption syndrome, especially zinc deficiency, may be associated with the development of sarcopenia [41,42,43].

### 3.2. Chronic Inflammation

Chronic inflammation is thought to play an important role in the development of sarcopenia in IBD. Patients with IBD are accompanied by a systemic increase in pro-inflammatory cytokines such as interferon (IFN) γ, IL-1, IL-6, and especially tumor necrosis factor (TNF) α [44]. Inflammatory cytokines are associated with protein catabolism and reduced muscle protein synthesis [44], while TNFα induces muscle protein degradation by inhibiting the anabolic mTORC1 pathway. In addition, the stimulation of muscle atrophy F-box (MAFBx) and muscle RING finger 1 (MURF-1) by TNFα promotes muscle protein degradation [45]. TNFα also increases reactive oxidative stress, activates the nuclear factor kappa-light-chain-enhancer of the activated B cells (NF-κB) pathway, and induces further downstream inflammation [46]. Inflammatory cytokines are known to activate NF-κB and the ubiquitin–proteasome system, as well as to reduce the levels of insulin-like growth factor-1 (IGF-1) in the plasma and muscle [47]. Decreased IGF-1 results in decreased activation of the phosphoinositide 3-kinase (PI3K) pathway, reduced stimulation of mTORC1, and decreased muscle protein synthesis. NF-κB and TNF-α induce myostatin, an inhibitor of muscle protein synthesis, and myostatin inhibits mTORC1 and activates MURF-1 and MAFBx [48]. In pediatric patients with IBD, an association between low serum IGF-1 levels and decreased QOL has been noted [47]. In patients with CD, the association between muscle fatigue and low serum IGF-1, high IL-6, etc. has been observed [49], and IGF-1 is inversely associated with disease activity [50].

### 3.3. Role of Adipose Tissue

It is known that the adipokines secreted by adipocytes play an essential role in the homeostasis of energy metabolism, but they are also involved in the regulation of the immune system and in the homeostasis of muscle proteins [51]. It has been known for a long time that in patients with CD, increased mesenteric adipose tissue is observed at a high frequency just below the ulcer lesion, and more visceral fat, especially mesenteric fat, has been observed on CT scans in CD than in UC [52]. The term “creeping fat” refers to the fat in the mesentery that occupies more than half of the area surrounding the inflamed bowel [52]. In patients with IBD, mesenteric fat does not necessarily correlate with body mass index (BMI), as it can increase with increased inflammatory status, even in the absence of elevated BMI, and thus an increase in mesenteric fat is often overlooked in patients with IBD and normal or low BMI. It is important to note that in patients with IBD and sarcopenia, where BMI is apparently preserved due to increased fat, loss of muscle mass may be overlooked [53]. Although BMI is a globally accepted screening tool for assessing nutritional status, it may not be a reliable indicator of malnutrition in patients with IBD. In a recent review by Adams et al. [54], 49% of the study cohort with IBD had sarcopenia, while 41.4% of these patients had normal weight and 19.5% were overweight or obese. In addition, adipocytes in the mesentery are directly involved in the induction of inflammatory responses in intestinal epithelial cells [55]. Adipocytes produce pro-inflammatory cytokines, such as free fatty acids, TNFα, and IL-6, and these cytokines are known to induce inflammatory responses by activating transcription factors, such as NF-κB, and signal transducer and activator of transcription 3 (STAT3) [55]. Inflammation of adipose tissue is associated with the exacerbation of IBD and may be involved in the development of sarcopenia.

### 3.4. Vitamin D Deficiency

Much attention has been paid to the role of vitamin D in patients with sarcopenia [56]. Vitamin D is fat-soluble and plays many important roles in skeletal muscle, including maintaining muscle contractile excitability via intracellular calcium, the proliferation and differentiation of skeletal muscle stem cells, and the maintenance of muscle function [56]. Vitamin D deficiency has been correlated with the risk of diseases such as sarcopenia, cardiovascular disease, obesity, osteoporosis, and cancer [57]. Vitamin D deficiency is more common in patients with IBD with low lipid intake or with malabsorption, and 30–47% of patients with IBD have been shown to have vitamin D deficiency [58]. A link between vitamin D receptor (VDR) and muscle protein anabolic signaling has been reported as the mechanism by which vitamin D deficiency is associated with muscle loss [59]. In addition to its direct effects on skeletal muscle, VDR is known to affect mitochondrial function, and decreased VDR results in decreased oxidative phosphorylation [60]. Mitochondrial dysfunction increases the production of reactive oxygen species that adversely affect skeletal muscle and can lead to sarcopenia [61]. Vitamin D plays a role in maintaining the homeostasis of the mucosal barrier by modulating immune responses, and vitamin D deficiency may increase susceptibility to mucosal damage, worsen the disease status of IBD, and induce sarcopenia [62]. Pediatric patients with CD and vitamin D deficiency are at high risk of sarcopenia [24]. Vitamin D deficiency is also a risk for osteosarcopenia [63]. On the other hand, in patients with UC, where the appearance of damaged lesions in the duodenum and small intestine is rare, vitamin deficiency is less frequent than in CD [64]. However, the absorption of vitamin D may be reduced due to interaction with 5-ASA preparations [65]. In patients with IBD, urinary excretion of calcium is enhanced during corticosteroid administration, increasing the risk of osteoporosis, and increased protein catabolism contributes to sarcopenia [65]. When IBD is active, the fat-soluble vitamins, vitamin A and vitamin E are decreased, as well as vitamin D [66]. Decreased serum levels of vitamins A and E have also been found in 16% of patients with CD [67]. A recent meta-analysis showed the benefit of vitamin D preparations in patients with UC [66].

A report followed 965 patients with IBD (61.9% with CD and 38.1% with UC) for 5 years; classified them into low and normal groups, according to their mean serum 25-hydroxyvitamin D levels during follow-up; and examined their relationship with drug use, medical care utilization, inflammatory markers, pain, and disease activity scores [68]. A total of 29.9% of the patients were classified as a low vitamin D group, and those in the low vitamin D group had a significantly higher frequency of corticosteroids, biologics and analgesics, CT scans, emergency visits, hospitalization, and need for surgery than those in the normal vit-D group (all *p* < 0.05). There was also a significant worsening of pain, disease activity score, and health-related quality of life (QOL) in the low vitamin D group (all *p* < 0.05) [68]. Vitamin D deficiency in patients with IBD is closely associated with clinical outcomes. On the other hand, a correlation between vitamin B12 deficiency and sarcopenia in the elderly has been reported, but not in patients with IBD [69].

### 3.5. Muscle–Gut Axis

The muscle–gut axis plays an essential role in skeletal muscle homeostasis [70]. A healthy gut microbiota regulates the immune homeostasis, metabolic homeostasis, and gene expression through the production of short-chain fatty acids (SCFAs) and antioxidants and allows immunocompetent cells in the mucosa to produce pro-inflammatory cytokines [70]. The gut microbiota has a direct influence on the bioavailability of amino acids [71], and some gut bacteria are derived from oral intake, which plays an essential role in the quantity and diversity of the gut microbiota [71]. The gut microbiota transmits nutrient signals and produces mediators for skeletal muscle homeostasis. SCFAs act on skeletal muscle mitochondria and affect muscle protein synthesis [72]. On the other hand, more than 99% of intestinal bacteria belong to the four phylums: *Firmicutes*, *Bacteroidetes*, *Proteobacteria*, and *Actinobacteria* [73]. There are several bacteria that have been identified to play a potential role in skeletal muscle function [74]. For example, *bifidobacteria* (*Actinobacteria* phylum) promote the breakdown of proteins into amino acids in the gut, produce SCFAs for energy production, stimulate the IGF-1/mTORC1 pathway, and promote the expression of genes involved in muscle protein synthesis [75]. In conditions with a reduced quantity and diversity of gut microbiota (i.e., dysbiosis), increased intestinal permeability leads to elevated myostatin in muscle and induces sarcopenia [76]. Patients with IBD have a high incidence of dysbiosis [77]. In patients with UC, the production of inflammatory cytokines such as TNFα can be enhanced by the invasion of *Fusobacterium varium* (*F. varium*), a commensal bacterium, and this has been suggested to be related to the development of UC [78]. *F. varium* has the ability to adhere to, and invade, colonic mucosal cells [78], and elevated pro-inflammatory cytokines caused by *F. varium* may be associated with the development of sarcopenia. Antibody titers to *F. varium* in patients with UC are significantly higher than those in CD and healthy individuals [79]. In contrast, CD-associated *Escherichia coli* with pro-inflammatory properties include adhesive invasive *Escherichia coli* (AIEC). It has been shown that AIEC is increased in approximately 38% of patients with active CD, compared to 6% of healthy individuals [77].

**Figure 1:**
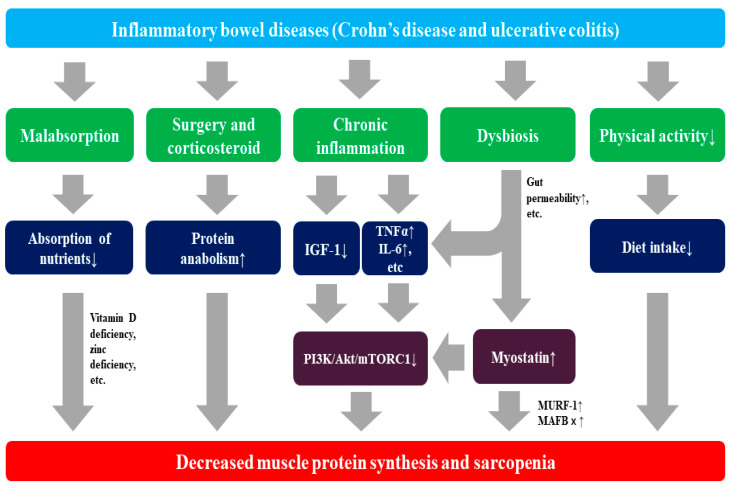
Mechanism for sarcopenia in patients with inflammatory bowel disease (IBD). IBD can cause sarcopenia through a variety of pathologies and immune responses. TNFα: tumor necrosis factor α, IGF-1: insulin-like growth factor 1, mTORC1: mechanistic target of the rapamycin complex 1, IL-6: interleukin-6, PI3K: phosphoinositide 3-kinase, MURF-1: muscle RING finger 1, MAFB x: muscle atrophy F-box.

The mechanism by which patients with IBD develop sarcopenia is shown in Figure 1.

## 4. Prognostic Impact of Sarcopenia in Patients with IBD

Table 2 shows reports on the relationship between sarcopenia and prognoses in patients with IBD [19,23,26,27,30,31,32,54,80,81,82,83,84,85,86,87]. There have been numerous reports that sarcopenia is a poor prognostic factor in patients with IBD undergoing surgery [19,26,27,30,31,54,81,82,83,84,85,86]. There are also reports that sarcopenia correlates with the severity of IBD [31,80]. There are reports that sarcopenia is helpful in determining treatment decisions for patients with IBD [23], that skeletal muscle adiposity is associated with prolonged hospitalization and rehospitalization in patients with IBD [83], and that sarcopenia is associated with osteoporosis in patients with IBD [87]. It has been reported that sarcopenia is a risk factor for the development of nonalcoholic fatty liver disease (NAFLD) in patients with IBD [88]. Kang et al. reported that among 443 patients with IBD, NAFLD was found in 11.1% and sarcopenia in 34.9%, with a higher incidence of sarcopenia in patients with NAFLD (51.0%), compared to patients with non-NAFLD (33.0%, *p* = 0.019). In their multivariate analysis, metabolic syndrome (hazard ratio (HR) = 8.63), small bowel resection (HR = 3.45), hyperuricemia (HR = 4.66), and sarcopenia (HR = 2.99) were independent adverse predictors for NAFLD in patients with IBD [88]. The prevalence of obesity in patients with IBD is reported to be 15–40% [89]. On the other hand, the number of elderly onset patients with UC has been increasing in recent years [90,91]. In an aging society, frailty is an important social problem, as well as sarcopenia [92]. Like sarcopenia, frailty also affects the prognosis of patients with IBD, such as rehospitalization, death, and severe infections [93,94,95,96]. The comprehensive assessment of physical frailty, cognitive frailty, and social frailty in elderly patients with IBD seems to be important for the current situation, where the number of elderly patients with IBD is increasing [96,97].

## 5. Intervention for Patients with IBD and Sarcopenia

### 5.1. Nutritional Intervention

There is increasing evidence to support nutritional interventions for maintaining muscle mass and increasing muscle strength and function in elderly people with sarcopenia [98]. An important nutrient to counteract sarcopenia is dietary protein, and in patients with IBD, a protein intake of 1.2–1.5 g/kg/day is often recommended during the active phase of IBD [99]. As mentioned earlier, STRIDE-II advocates the avoidance of structural destruction and dysfunction of the intestinal tract, and improvement of physical function and QOL or ADL as long-term therapeutic goals for patients with IBD, so nutritional intervention in patients with IBD and sarcopenia is highly significant [16]. However, while optimizing nutritional status may be an effective countermeasure against sarcopenia in IBD, there is currently no established evidence on the role of specific diets or supplements for the prevention and treatment of sarcopenia in IBD. On the other hand, recent advances in nutritional therapy for postoperative short bowel syndrome for Crohn’s disease can inhibit the exacerbation of sarcopenia [100].

### 5.2. Exercise

Exercise, both aerobic and resistance, is a well-established way to improve muscle mass and strength [101]. Physical activity is now recognized as having an anti-inflammatory effect [102]. Patients with IBD often suffer from lack of physical activity, and thus physical exercise for patients with IBD could be used as supportive intervention [103]. However, unlike healthy individuals, there are concerns about exercise intervention for patients with IBD. Although there are a number of studies that demonstrated the safety and efficacy of exercise interventions in patients with IBD, these studies have often been conducted in patients with IBD with mild disease status or in remission, and a thorough evaluation is required to determine whether exercise interventions in patients with active IBD are an aggravating factor for IBD [103,104,105,106,107,108,109]. The significance of exercise intervention for improving sarcopenia in patients with IBD with higher disease activity is unknown [104,105,106,107,108,109].

### 5.3. Pharmacological Intervention

There have been several reports on drug interventions in patients with IBD and sarcopenia. Infliximab improves sarcopenia in patients with CD [110]. In a study of 19 patients with CD, both muscle mass and muscle strength improved significantly after 24 weeks of infliximab treatment, and IL6 was significantly reduced [110]. Loss of skeletal muscle mass correlates with UC activity and improves after pharmacological therapy and colorectal surgery [31]. Several studies have demonstrated the benefits of vitamin D supplementation in patients with IBD, but only a limited number of studies have demonstrated the effects of vit-D supplementation on muscle mass and function. While, vitamin D supplementation improves muscle strength in presarcopenic elderly people [111], healthy people between the ages of 18 and 40 [112], and postmenopausal women [74]. There are reports that vitamin D supplementation in pediatric patients with IBD improved bone mineral density and muscle strength [113], but data in adult patients with IBD are lacking. Vitamin D supplementation in patients with IBD also improves dysbiosis [114]. VDRs are involved in the regulation of T cell and Paneth cell function and regulate the release of antimicrobial peptides in gut bacteria–host interactions [114]. On the other hand, mucosal healing and disease remission in patients with IBD can significantly improve oral feeding and nutritional status, and prevent muscle loss. It should also be noted that biologics such as anti-TNF-α antibodies, anti-α4β7 integrin antibodies, and Janus kinase (JAK) inhibitors improve the mucosal healing rate for moderate to severe UC, allowing early feeding and contributing to the improvement of sarcopenia [7,9]. Table 3 summarized the results of interventions for patients with IBD, focusing on sarcopenia. The number of reported studies is small.

## 6. Final Remarks

In this review, IBD and sarcopenia were outlined. Although IBD is more common in younger patients, sarcopenia can develop through a variety of mechanisms, including malnutrition and chronic inflammation. It should be noted that the prevalence of patients with IBD is increasing significantly worldwide, and a certain number of these patients have sarcopenia. It should also be noted that the number of elderly patients with UC who are prone to the complications of sarcopenia and/or frailty is increasing. The standard values of the evaluation items (grip strength, muscle mass, etc.) for determining sarcopenia in patients with IBD have not been established, and this is an issue for the future. Evidence is accumulating that sarcopenia is a prognostic factor in patients with IBD, and appropriate intervention for sarcopenia may be important, in addition to clinical remission and endoscopic mucosal healing. In particular, there is an urgent need to establish exercise therapy in patients with IBD and sarcopenia. It is hoped that more evidence will be collected on interventions for patients with IBD and sarcopenia.

## Figures and Tables

**Table 1:** Prevalence of sarcopenia in adult or pediatric patients with inflammatory bowel diseases reported in recent years.

Author [Ref.]	Country	Year	Disease	*N*	Age (Mean, Median or Range)	Assessment Modality	Sarcopenia (%)
Ünal et al. [17]	Turkey	2021	UC or CD	344	49	EWGSOP2	41.3
Boparai et al. [18]	India	2021	CD	44	34	CT (L3)	43
Ge et al. [19]	China	2021	Acute severe UC	233	43.6	CT (L3)	50.2
Celentano et al. [20]	UK	2020	CD	31	46	MRI (L3)	38
Lee et al. [21]	Korea	2020	CD	79	29	CT (L3)	50
Grillot et al. [22]	France	2020	CD	88	35	CT (L3)	58
Cushing et al. [23]	USA	2018	Acute severe UC	89	43	CT (L3)	70
Mager et al. [24]	Canada	2018	Pediatric UC or CD	85	5 to 18	DXA	23.5
Thiberge et al. [25]	France	2018	CD	149	41	CT (L3)	33.6
Dedhia et al. [26]	USA	2018	Pediatric UC	29	15.9	MRI (L3)	62
Fujikawa et al. [27]	Japan	2017	UC	69	39.8	CT (L3)	25.9
Csontos et al. [28]	Hungary	2017	CD	126	34	BIA	29.4
Holt et al. [29]	Australia	2017	CD	44	38	CT or MRI (L3)	41
Bamba et al. [30]	Japan	2017	CD	43	29	CT (L3)	37
Zhang et al. [31]	China	2017	UC or CD	204	25	CT (L3)	UC: 27.3 CD: 59.0
Cravo et al. [32]	Portugal	2017	CD	71	43	CT (L3)	31

UC, ulcerative colitis; CD, Crohn’s disease; EWGSOP2, European Working Group for Sarcopenia in Older People 2; CT, computed tomography; MRI, magnetic resonance imaging; DXA, dual-energy X-ray absorptiometry; BIA, bioelectrical impedance analysis.

**Table 2:** Prognostic impact of sarcopenia in patients with inflammatory bowel disease.

Author [Ref.]	Country	Year	*N* (Disease)	Notable Findings
Zager et al. [82]	Israel	2021	121 (CD)	Psoas muscle area is an easily measured radiological parameter linked to postoperative complications in patients with CD receiving bowel resection.
Ge et al. [19]	China	2021	233 (UC)	Sarcopenia is helpful for predicting the clinical course and postoperative outcomes in patients with acute severe UC.
Atlan et al. [80]	Israel	2021	32 (UC), 69 (CD)	Sarcopenia involves significant correlation with the severity of pediatric IBD and serves as a predictor for adverse clinical disease outcome.
Bamba et al. [81]	Japan	2020	99 (CD), 88 (UC)	Low muscle volume and low visceral adipose tissue volume negatively affect the long-term outcome of intestinal resection.
Galata et al. [83]	Germany	2020	230 (CD)	SMI was the only significant adverse factor for Clavien–Dindo complications grade ≥ III.
Dedhia et al. [26]	USA	2018	29 (UC)	Paraspinous muscle area on MRI is associated with complications and increased hospital stay after colectomy in patients with pediatric UC.
Cushing et al. [23]	USA	2018	89 (UC)	Sarcopenia, as determined on abdominal CT, was a novel predictor of need for rescue therapy in hospitalized patients with UC.
Stephen et al. [84]	Ireland	2018	21 (UC), 52 (CD), 4 (Im)	Myosteatosis was associated with prolonged hospital stay and increased 30-day hospital readmission rates in a multivariate analysis.
Adams et al. [54]	USA	2017	14 (UC), 76 (CD)	Sarcopenia was a predictor for surgical resection in patients with IBD with a body mass index ≥25 kg/m^2^.
Pederson et al. [85]	USA	2017	51 (UC), 127 (CD)	In IBD patients younger than 40 years, sarcopenia affects surgical outcomes.
Fujikawa et al. [27]	Japan	2017	69 (UC)	Sarcopenia is a predictor for surgical site infection after pouch surgery in patients with UC.
Zhang et al. [31]	China	2017	99 (UC)	SMI correlated significantly with UC disease activity. Sarcopenia (HR = 8.49, *p* = 0.007) was an adverse independent predictor of high Mayo score in patients with UC. Patients with UC and sarcopenia had a high probability of need for colectomy.
Bamba et al. [30]	Japan	2017	29 (UC),43 (CD)	The cumulative operation-free survival rate was significantly lower in patients with IBD and sarcopenia than in all patients with IBD.
Cravo et al. [32]	Portugal	2017	71 (CD)	A lower muscle attenuation and a high visceral fat index were associated with disease severity in patients with CD.
Zhang et al. [86]	China	2017	114 (CD)	The prevalence of sarcopenia is higher in patients with CD requiring bowel resection. It significantly elevates the risk of major postoperative complications.
Bryant et al. [87]	Australia	2015	42 (UC), 95 (CD)	Low lean mass and sarcopenia are common in patients with IBD and important to recognize, as they predict osteopenia or osteoporosis.

UC, ulcerative colitis; CD, Crohn’s disease; Im, Intermediate; IBD, inflammatory bowel disease; CT, computed tomography; MRI, magnetic resonance imaging; SMI, skeletal muscle index; HR, hazard ratio.

**Table 3:** The results of interventions for IBD patients focusing on sarcopenia.

Author [Ref.]	Year	Disease	*N*	Intervention	Outcome
Sigurdsson et al. [107]	2021	Young IBD	41	Physical exercise	Physical exercise is associated with beneficial bone mineral density and body composition in young patients with IBD.
Seeger et al. [108]	2020	Mild CD	45	Physical exercise	The maximal and average strength in the upper and lower extremities increased significantly
Jones et al. [109]	2020	Stable CD	47	Physical exercise	The exercise group had superior values for all muscle function outcomes and lower fatigue severity.
Hradsky et al. [113]	2017	Pediatric IBD	55	2000 IU of Cholecalciferol	Cholecalciferol substitution was positively associated with trabecular bone mineral density and maximal muscle power.
Subramaniam et al. [110]	2015	CD	19	infliximab	Muscle mass and muscle strength improved significantly.

IBD, inflammatory bowel disease; CD, Crohn’s disease.
